# Analysis of G-quadruplex forming sequences in podocytes-marker genes and their potential roles in inherited glomerular diseases

**DOI:** 10.1016/j.heliyon.2023.e20233

**Published:** 2023-09-15

**Authors:** Mona Saad, Cybel Mehawej, Wissam H. Faour

**Affiliations:** a Gilbert and Rose-Marie Chagoury School of Medicine, Lebanese American University, Byblos, Lebanon; b Department of Human Genetics, Gilbert and Rose-Marie Chagoury School of Medicine, Lebanese American University, Byblos, Lebanon

**Keywords:** G-quadruplex, Podocytes-marker genes, Nephrotic syndrome, Single nucleotide polymorphisms (SNPs), Sequence analysis

## Abstract

Nephrotic Syndrome is the most widespread pediatric kidney disorder. Genetic alterations in podocyte genes are thought to be responsible for the disease. G-quadruplexes are non-conventional guanine-rich DNA and RNA structures, which are commonly found in regulatory regions. This study examined the potential G-quadruplexes forming sequences in the promoters and gene bodies of podocyte-marker genes. High G-quadruplexes density was found in the *vascular endothelial growth facto*, cluster of differentiation-151, integrin subunit beta-4, metalloendopeptidase, Wilms tumor-1, integrin subunit beta-3, synaptopodin, and nephrin promoters. *Vascular endothelial growth facto*, cluster of differentiation-151 and integrin subunit beta-4 had the highest G-quadruplexes density in their gene bodies and promoters. Additionally, highly stable G-quadruplexes forming sequences were identified within all podocyte-marker genes. Furthermore, it is hypothesized that Wilms tumor-1 is capable of controlling the transcription of podocalyxin by binding to two possible G-quadruplexes forming motifs. We next analyzed the most frequently reported genetic mutations in the selected genes for their effect on DNA G-quadruplexes formation, and the thermodynamic stability of predicted RNA G-quadruplexes, using RNAfold. Importantly, the missense mutation c.121_122del in the nephrin gene reported in patients with NS type 1 affected DNA G-quadruplexes formation in this region as well as the thermodynamic stability of the corresponding RNA G-quadruplexes. Overall, we report the potential regulatory roles of G-quadruplexes in the etiology of nephrotic syndrome and their possible use as drug targets to treat kidney diseases.

## 1 Introduction

Nephrotic syndrome (NS) is a heterogeneous disorder characterized clinically by the presence of edema and increased glomerular filtration barrier permeability for macromolecules leading to nephrotic-range proteinuria, hypoalbuminemia and hyperlipidemia [1].

Podocytes are key cells involved in the formation and the maintenance of a functional glomerular barrier. Damage to podocytes is associated with a plethora of kidney dysfunction and albuminuria [2]. Several genes are involved in podocytes health and mutation in any of these genes causes glomerular damage and proteinuria. Among these, neprin (*NPHS1)* [3]*,* podocin *(NPHS2)* [4]*,* alpha-actinin-4 *(ACTN4)* [5]*,* CD-2 associated protein *(CD2AP)* [6]*,* podocalyxin *(PODXL)* [7], synaptopodin *(SYNPO)* [8]*,* Wilms tumor *(WT1)* [9]*,* metalloendopeptidase *(MME)* [10]*,* protein tyrosine phosphatase receptor type O *(PTPRO)* [11]*, vascular endothelial growth factor-A (VEGFA)* [12]*,* integrin subunit beta-1 *(ITGB1)* [13]*,* integrin alpha-3 *(ITGA3)* [14]*,* integrin subunit beta 3 *(ITGB3)* [15]*,* integrin subunit alpha V *(ITGAV)* [16]*,* integrin subunit alpha-6 *(ITGA6)* [17]*,* integrin subunit beta-4 *(ITGB4)* [18] and cluster of differentiation 151 *(CD151)* [19]*. NPHS1* encodes the nephrin protein, a cell adhesion and a structural molecule of the glomerular filtration barrier, located in the slit diaphragm of the glomerular podocytes. *NPHS2* encodes podocin**,** a member of the stomatin family that plays a key role in the regulation of the glomerular permeability. *PODXL* is a member of the sialomucin protein family originally identified as an important component of glomerular podocytes**.** *ACTN4, CD2AP, PODXL* and *SYNPO* are actin-binding proteins. *WT1* is a transcription factor having essential role in the normal development of the urogenital system. *PTPRO* is a member of the R3 subtype family of receptor-type protein tyrosine phosphatases that regulates glomerular pressure and permselectivity [20]. *VEGFA* is a member of the PDGF/VEGF growth factor family that induces proliferation and migration of vascular endothelial cells, and is essential for both physiological and pathological angiogenesis. *ITGA3, ITGA6, ITGAV, ITGB1, ITGB3* and *ITGB4* are members of the integrin family that participate in cell adhesion as well as cell-surface mediated signaling [17]. *CD151* is a member of the tetraspanin family that is known to complex with integrins regulating its trafficking and/or function. The best-characterized nephrotic syndromes are the congenital nephrotic syndrome of the Finnish type (CNF) (MIM#256300) and the steroid resistant nephrotic syndrome (SRNS) (MIM#600995). They are caused by mutations in *NPHS1* and *NPHS2* genes, respectively [1,17].

G-quadruplexes (G4s) are non-canonical DNA and/or RNA secondary structures that may form in guanine-rich regions. They are characterized by the stacking of several G-quartets, where every G-quartet is a tetrad formed by the association of four guanine residues linked through Hoogsteen Hydrogen bonds and stabilized by monovalent cations, predominantly K + [21]. G4s are generally defined by the sequence G_≥2_N_1-7_G_≥2_N_1-7_G_≥2_N_1-7_G _≥ 2_; whereby G represents guanines in the tract and N represents the nucleotide loop length. G-quadruplexes are not distributed randomly in the genome but they are located in specific regions such as the promoters of oncogenes like *MYC* [22] and *KRAS* [23]*,* in telomeres [24], in immunoglobulin switch regions [25] and in ribosomal DNA [26]. Stable RNA G-quadruplexes (G4s)were also documented in 5’ UTRs [27], some introns [28], non-coding RNAs [29], and in open reading frames (ORF) [30]. Since G-quadruplexes are stable under physiological conditions, their presence may represent a knot in the genome affecting replication, transcription and translation. In transcription, it is thought that G4s can induce or inhibit transcription according to the localization of G4 motif in the template or the non-template strand. Additionally, proteins acting as transcription factors or repressors can bind to G4 motifs and affect DNA transcription [31]. In translation, emerging evidence support the role of G-quadruplexes in pre-mRNA splicing, polyadenylation, mRNA targeting and translation [32]. G4-quadruplex structures exist in the promoter of significant number of oncogenes and represent an attractive target for anticancer drug design. They are very polymorphic in topology, and deciphering their precise 3D structure is a key step to design G4-stabilizing ligands and thus to control gene expression [33]. The formation of G-quadruplexes is predicted *in silico* by using computational methods. Several algorithms were developed including Quadparser [34], QGRS Mapper [35] and others. “G4Hunter” is a new algorithm and was developed by “Bedrat et al., 2016” that took into account G-richness and G-skewness and provided a propensity score for every tested sequence [36].

To date, G-quadruplex structures were not reported in genes related to kidney diseases. The aim of this work was to study G-quadruplexes function in the genes involved in nephrotic syndrome. The above-mentioned podocyte-related genes were selected and analyzed for the presence of putative G4 forming sequences (PQSs) using G4Hunter and QGRS Mapper algorithms. Furthermore, we evaluated the most frequently reported genetic mutations in these genes found in patients with nephrotic syndrome. Then, we studied *in silico* the possible effect of these mutations on DNA G4 formation as well as on the thermodynamic stability of the predicted RNA G4s in either exons or introns containing these mutations. Our data suggests that glomerular diseases associated mutations could affect RNA G4 stability and/or DNA G4 formation in podocyte-marker/related genes which may be linked to the observed phenotype. Our study will provide a new tool to explore the roles of G4s in podocyte-marker genes’ regulation and help exploring novel treatments for glomerular diseases.

## 2 Results

### 2.1 Analysis of putative G4 forming sequences (PQSs) in podocytes-marker genes by G4Hunter and QGRS mapper

We first analyzed putative G4 forming sequences in selected podocytes-marker genes including ***NPHS1***, ***NPHS2***, ***ACTN4***, ***CD2AP***, ***PODXL***, ***SYNPO***, ***WT1***, ***MME***, ***PTPRO***, ***VEGFA***, ***ITGB1***, ***ITGA3***, ***ITGB3***, ***ITGAV***, ***ITGA*6**, ***ITGB4*** and ***CD151* (S1 Table)**.

**Fig. 1:**
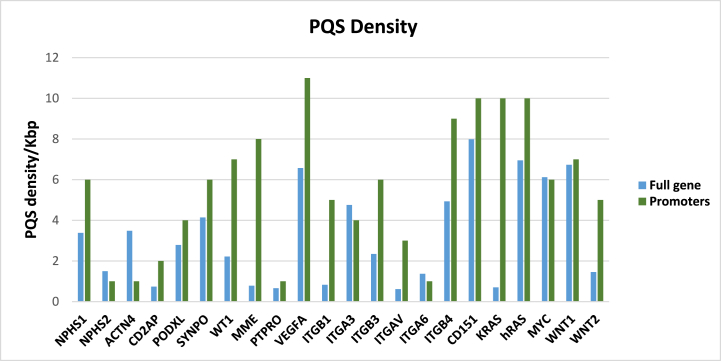
PQS density (number of G4/1000 bp) in gene bodies and promoters determined by using G4Hunter in the 17 podocytes-marker genes and the 5 oncogenes.

By using G4Hunter**,** putative G-quadruplex forming sequences (PQSs) were mapped in the gene bodies of the selected 17 podocytes-marker genes and in the upstream region including 1000 bp before their Transcription start sites (TSSs). G-quadruplex motifs are enriched in the promoters of oncogenes so five oncogenes (*KRAS, HRAS, MYC, WNT1* and *WNT2*) were added as control. We chose 1.2 as threshold and 25 as window size in our analysis since these parameters proved good accuracy when validated experimentally [36]. The frequency of PQS was also identified within the promoter regions and gene bodies of the selected genes (PQS/Kbp). *CD151, VEGFA* and *ITGB4* presented the highest G4 density overall, in their promoters and gene bodies compared to the other podocytes genes. In comparison with the oncogenes promoters, the podocytes genes VEGFA, CD151, ITGB4, MME, WT1, ITGB3, SYNPO, and NPHS1 exhibited high G4 density (Fig. 1). The same sequences were also reanalyzed using another G4 predicting tool called QGRS Mapper that predicts PQS according to a pattern-matching sequence [35]. We searched for PQS containing 2 ≥ G and a loop length between 0 and 36. Importantly, PQS with high scoring (containing a minimum of 3 G-tetrads) were documented in all podocyte-marker genes **(S2 Table).** These PQSs presented G4 scores between 37 and 84 reflecting their high stability.

We sought to further investigate if WT1 a known transcription regulator of both *NPHS1* and *PODXL* binds to putative G4 motifs [37] in these genes. Accordingly, we analyzed the genomic sequence of *NPHS1* for potential G4 forming sequences by including the region 1.2 kb before the TSS by using G4Hunter and QGRS Mapper. None of these two algorithms detected the WT1 binding site sequence (5′-GGTAGGAATGGAGGAGGAGGAG-3′). However, several sequences containing a repetition of the motif 5′-GGAGG-3′ were detected. Despite that, a score of 1.14 was attributed to this 22bp sequence by G4Hunter, suggesting possible folding into G-quadruplex structure. The G4Hunter tool might mistakenly classify the WT1 binding site sequence as a false negative.

Similarly, for the regulation of *PODXL* by WT1, we analyzed the genomic sequence of *PODXL* for potential G4 forming sequences by taking into account the region 1.3 kb before the TSS. G4Hunter detected two of the WT1 binding sites PCp1 (5′-GGTGGGAGTGGGTGT-3’; G4 score: 1.6) and PCp3 (5′-GCGGGGGCGGGGGCGGGGGCGGGGGCC-3’; G4 score: 2.7) demonstrating their folding into G4s at least *in silico.* As PCp1 has been demonstrated to be the main binding site for WT1 in PODXL [38], we suggest that WT1 could induce the transcription of PODXL by binding to the G4 sequence formed by PCp1 mainly and PCp3 to a less extent.

### 2.2 Mutations in podocytes-associated genes in kidney disease patients affect DNA G4 formation and the thermodynamic stability of RNA G4s

We aimed to determine the possible effect of mutations associated with nephrotic syndromes on DNA G4 formation and RNA G4s stability.

For this, we referred to the literature as well as OMIM to identify the most frequent mutations found in patients with NS in 17 podocytes-marker genes. Since we found that some mutations for some genes are not very frequent, we limited our study to *NPHS1, NPHS2, CD2AP, ACTN4, WT1, PTPRO, ITGA3* and *ITGB4* genes. Information about the selected mutations is summarized in **(**Table 1**)**.

**Table 1:** The most frequent mutations in *NPHS1, NPHS2, CD2AP, ACTN4, WT1, PTPRO, ITGA3* and *ITGB4* genes associated with nephrotic syndrome.

Gene	Mutation	Type of mutation	dbSNP Reference	Localization in the gene	Disease
*NPHS1*^a^	c. 121_122del, p.Leu41fs	Frameshift Deletion	rs386833873	Exon 2	Nephrotic syndrome, type 1
	c.3325C > T, p.R1109X	Nonsense	rs137853042	Exon 26	Finnish congenital nephrotic syndrome
*NPHS2*^b^	c.413G > A; p.R138Q	Missense	rs74315342	Exon 3	Nephrotic syndrome, type 2
	c.686G > A, p.R229Q	Polymorphism	rs61747728	Exon 5	Nephrotic syndrome, type 2
*CD2AP*^c^	g.47544259_47544260delinsCT; p.P243S	Splicing	rs1554181304	Exon 7	Focal segmental glomerulosclerosis 3
	c.1834C > T, p.R612X	Nonsense	rs267606710	Exon 17	Focal segmental glomerulosclerosis 3
*ACTN4*^d^	c.763 A > G; p.K255EGlu	Missense	rs121908415	Exon 8	Focal segmental glomerulosclerosis 1
	c.776C > T; p.T259I	Missense	rs121908416	Exon 8	Focal segmental glomerulosclerosis 1
	c.784T > C; p.S262P	Missense	rs121908417	Exon 8	Focal segmental glomerulosclerosis 1
*WT1*^e^	c. 1180C > T; p.R394W	Missense	rs1564972874	Exon 7	Nephrotic syndrome, type 4
*PTPRO*^f^	c.2627+1G > T; p.E854_W876del	Splicing	rs1591732280	Intron 16	Nephrotic syndrome, type 6
	c.2745+1G > A	Splicing	rs1591750243	Intron 18	Nephrotic syndrome, type 6
*ITGA3*^g^	c.1883G > C, p.R628P	Missense	rs140781106	Exon 14	Interstitial lung disease, nephrotic syndrome, and epidermolysis bullosa, congenital
	c.1387C > T; p.R463W	Missense	rs797044989	Exon 10	Interstitial lung disease, nephrotic syndrome, and epidermolysis bullosa, congenital
*ITGB4*^h^	c.3841C > A; p.R1281W	Missense	rs121912467	Exon 31	Epidermolysis bullosa junctionalis with pyloric atresia

a The two mutations of Fin-major (nt121delCT) and Fin-minor (p.R1109X) in *NPHS1* are highly prevalent (>90%) in the Finnish population and generally cause severe early-onset phenotypes [1].

b The p.R138Q mutation in *NPHS2* is the most frequently found pathogenic *NPHS2* mutation and is believed to be a founder mutation in Europe. p.R229Q is the most common *NPHS2* variant in people of European descent, with a frequency of between 2 and 3% in this population [2]. However, it has a high frequency in SRNS cases (5.3% in a study by Santin et al.) [3].

c The mutations *p.P243S and p*.R612stop in *CD2AP* are the most frequent pathogenic mutations in this gene.

d p.K255E, p.T259I, and p.S262P mutations are all located within the evolutionarily conserved ABD of ACTN4. All mutations are associated with increased ACTN4 binding affinity to F-actin [4].

e p.R394 mutation in *WT1* is selected because it is the most frequently found in 50% of the DDS patients [5].

f c.2627+1G > T and c.2745+1G > A are two selected mutations affecting splice donor variants in *PTPRO* and reported in OMIM database.

g p.R628P and p.R463W are the most frequent pathogenic mutations in *ITGA3* [6].

h p.R1281W is one of the most frequent and pathogenic mutations in *ITGB4* [7].

**Fig. 2:**
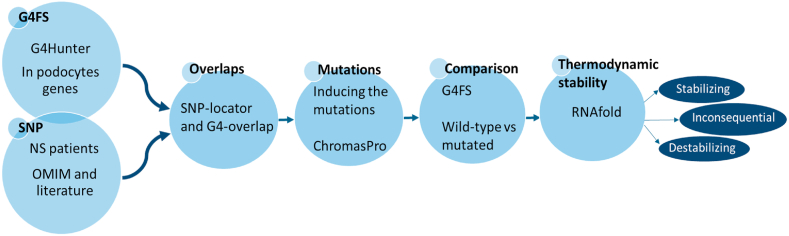
Flowchart illustrating the procedure for determining the possible effect of mutations associated with nephrotic syndrome on DNA G4 formation and RNA G4 stability. First, putative G4 forming sequences were identified in the genes bodies of the selected podocytes marker genes by using G4Hunter. In parallel, the most frequent mutations SNPs in these genes reported in patients with Nephrotic syndrome NS were identified by referring to the literature and OMIM database. Then, overlaps between the G4FS and the SNPs were determined by using our python codes SNP-locator and G4-overlap. The mutations in the genomic sequences were induced by using ChromsPro. Subsequently, the number of G4 sequences in the wild-type genomic sequences was compared to the one in the mutated states. Finally, by using RNAfold, the thermodynamic stability of RNA G4s in wild-type and mutated sequences was determined in the context of the G4-containing exon or intron and their flanking sequences. The effect of each mutation could result in G4 stabilization, G4 destabilization or inconsequential.

To that aim, we used our homemade python codes SNP-locator and SNP-overlap [39] in order to find the overlaps between the mutations and G4 sequences or to determine the nearest G4 sequence to the mutation in each gene body. After this step, we introduced the mutations to the sequence of each gene. We next compared the number of DNA G4 sequences predicted in the context of wild type and mutated genomic sequences to see if the mutation has an effect on the number of DNA G4 sequences or if there is any shift in the affected G4 sequence (Fig. 2). UCSC genome browser showed that the frameshift deletion c. 121_122del in *NPHS1* exon 2 **(localization: Chr 19: 36342511–363342512**) is located 1 bp before the G4 sequence (**GACGGTGGTGGAGGGGGCCTCAGTGGAG; Chr 19: 36342510**–**36342483, G4 score:1.18) (S1 Fig).** Importantly, this genetic deletion of 2bp changed the G4 sequence formed to **GAAAACGACGGTGGTGGAGGGGGCCTCAGTGGAG (Chr 19: 36342516**–**36342483, G4 score: 0.971)** by a shift of 6 bp leading apparently to a destabilization of the DNA G4 sequence formed in this position **(**Table 2**).** For all the other genes, the selected mutations do not overlap with any predicted DNA G4 sequence and none of these mutations changed PQS density, caused a shift in DNA G4 sequences or affected their stability **(**Table 2**).**

**Table 2:** Table illustrating the mutations selected, their localization, the DNA G4 sequence implicated, the strand, the localization of the G4 sequence and the presence or absence of shift in DNA G4.

	Mutation details		G4 sequence details			
Gene	Mutation	Localization	DNA G4 Sequence (5′-3′)	Strand	Localization	Shift
*NPHS1*	c. 121_122del, p.L41fs	Exon 2	GACGGTGGTGGAGGGGGCCTCAGTGGAG/GAAAACGACGGTGGTGGAGGGGGCCTCAGTGGAG **(a)**	positive	Exon 2	Yes
	c.3325C > T, p.R1109X	Exon 26	GGATGAAGGTGTGGGGGGAAGTTGAGTGCTG	negative	Intron 25	No
*NPHS2*	c.413G > A; p.R138Q	Exon 3	GAAAGGTTTAGTAGTGGGGGTTTGGAG	positive	Intron 3	No
	c.686G > A, p.R229Q	Exon 5	GCTGGCAGGAACGGTGGGGTTGGTGGGGATGGACAGGAGGGGTTGG	positive	Intron 5	No
*CD2AP*	g.47544259_47544260delinsCT; p. P243S	Exon 7	GGTTGGGGGTGGGGGGCAG	negative	Intron 8	No
	c.1834C > T, p.R612X	Exon 17	GGAAGAGGGGGGGAATAGGATTAAGGAAAGAGGG	positive	Intron 16	No
*ACTN4*	c.763 A > G; p.K255E	Exon 8	GCCCGAGTAGGGCGGGGGGTGGAGGCGTCACTCACTGGG	negative	Intron 7	No
	c.776C > T; p.T259I	Exon 8	GCCCGAGTAGGGCGGGGGGTGGAGGCGTCACTCACTGGG	negative	Intron 7	No
	c.784T > C; p.S262P	Exon 8	GCCCGAGTAGGGCGGGGGGTGGAGGCGTCACTCACTGGG	negative	Intron 7	No
*WT1*	c. 1180C > T; p.R394W	Exon 7	GGGGATCTGGAGTGTGAATGGGAGTGG	positive	Intron 6	No
*PTPRO*	c.2627+1G > T; p.E854_E876del	Intron 16	GGAGATGCTGGGTTGGCATGGGGGCTCAGGCCTG	negative	Intron 16	No
	c.2745+1G > A	Intron 18	GGAAAGGGCCTGGGAATTGGGGGGTTTGTGTTGCGGTCATG	positive	Intron 18	No
*ITGA3*	c.1883G > C, p.R628P	Exon 14	GGAACTGGACCTGGGGGGTGGCCGGAGGTGTGAAGG and GGCCAAGGGTGGGACGGGGCCTCATTAACTGGCAGGGTGGGGGCGGGGCCTCATGGCAAGGCGAG	negative and positive	Intron 13-exon 14/intron 14	No
	c.1387C > T; p.R463W	Exon 10	GGGAGGCTAGGAGGGGCTGCAG	positive	Intron 10	No
*ITGB4*	c.3841C > A; p.R1281W	Exon 31	GGCGCGCAACGGGGCCGGCTGGGGGCCTGAGCGGGAGG	positive	Exon 31	No

(a) The G4 sequence obtained after the deletion.

Since these mutations affect almost the exonic regions and few affect the intronic regions, we hypothesized that these mutations could influence the stability of the corresponding RNA G4 sequences in these regions. According to G4Hunter, the corresponding RNA sequence for each DNA G4 sequence located in the sense strand can fold into G4 structure [36]. For this, we compared the thermodynamic stability of the RNA G4s represented by ΔG° (kcal/mol) in the wild type and mutated sequences in the context of exons or introns containing G4 and their flanking sequences by using the RNAfold software (Fig. 2).

The c. 121_122del mutation in *NPHS1* increased the minimum free energy ΔG° from −163.9 to −164.3 kcal/mol demonstrating an enhancement in the stabilization of the RNA G4 in this region **(S3 Table)**. Other than c. 121_122del mutation, the mutations p.R229Q in *NPHS2*, p.K255E and p.S262P in *ACTN4* and p.R628P in *ITGA3* provoked an increase in the thermodynamic stability of RNA G4 sequences. While, the mutations p.R1109X in *NPHS1*, p.R138Q in *NPHS2*, p.R612X in *CD2A*P, p.T259I in *ACTN4*, p.R394W in *WT1*, p.Glu854_Trp876del, c.2745+1G > A in *PTPRO* and p.R1281W in *ITG*B4 provoked a destabilization of RNA G4 sequences in these regions. However, the mutations p.R463W in *ITGA3* and p.P243S in *CD2AP* did not affect the stability of RNA G4s in their context.

As stated above, this deletion of 2bp in *NPHS1* exon 2 occurring just before the RNA G4 sequence (5′-GACGGUGGUGGAGGGGGCCUCAGUGGAG-3′) provokes a stabilization of the RNA secondary structure in this location. The observed severe phenotype in these patients could be related to the fact that the resulted stabilization of the RNA G4 decreases the translation efficiency of *NPHS1* leading to the absence of expression of this gene.

## 3 Discussion

Glomerular podocytes play an important role in plasma ultrafiltration. Mutations in podocytes genes are involved in NS pathogenesis. Accumulated studies have demonstrated G-quadruplexes’ potential roles in regulating multiple biological functions including regulation of transcription, translation, DNA replication, and RNA localization [40]. In this work, we studied computationally for the first time potential G4 forming sequences in the promoters and gene bodies of 17 podocytes-marker genes and we demonstrated the presence of high density of G4s in *VEGF, CD151, ITGB4, MME, WT1, ITGB3, SYNPO* and *NPHS1* in comparison with the oncogenic promoters, with the highest G4 density in the gene bodies and promoters of *CD151, VEGFA* and *ITGB4* genes among the other selected podocytes genes. The latter finding corroborated the results obtained for cancer genes used as control in our study. We then, analyzed PQSs by using the two algorithms G4Hunter and QGRS Mapper that differ by the way they search for predicted G4s. Importantly, QGRS Mapper predicted PQSs with high score (between 37 and 84) in almost all the selected podocytes genes reflecting high stability and subsequent high probability to form G4 structures. The presence of such structures in the selected podocytes marker genes suggests strongly important roles of G-quadruplexes in the regulation of gene expression that must be further investigated.

WT1 is a transcription factor known to regulate the transcription of *NPHS1* and *PODXL* genes [37,38]. Wagner and colleagues [41] identified a WT1 responsive element 5′-GGTAGGAATGGAGGAGGAGGAG-3′ in *NPHS1* promoter. Moreover, published binding sites for WT1 have been demonstrated to be GC rich or contain TCC (opposite strand GGA) motifs [37]. While, G4Hunter and QGRS Mapper did not detect the 22 bp binding site sequence, several sequences containing a repetition of the motif 5′-GGAGG-3′ were detected. Although this sequence was not predicted to fold into G4 structure *in silico*, we suggest that it could be leaked as a false negative hit since a G4 score of 1.14 was assigned to it. Similarly to *NPHS1*, Palmer and colleagues [38] identified three potential binding sites in *PODXL* demonstrating a primary role of PCp1 binding site in the activation of the *PODXL* promoter. By re-analyzing the genomic sequence of *PODXL* for potential G4 forming sequences, G4Hunter detected PCp1 (5′-GGTGGGAGTGGGTGT-3’; G4 score:1.6) and PCp3 (5′-GCGGGGGCGGGGGCGGGGGCGGGGGCC-3’; G4 score: 2.7) demonstrating that the two binding sites PCp1 and PCp3 of *PODXL* fold into G-quadruplexes. Our results highlighted the important role that could be played by G4 structures in the regulation of *NPHS1* and *PODXL* transcription by WT1 and their effects on normal podocytes markers expression.

Genetic mutations in podocytes-marker genes contributing to NS are well established [17]. “Baral et al., 2012” demonstrated that there is a strong association between single nucleotide polymorphisms located in predicted G4 sequences and expression of the corresponding genes in individuals in a genome-wide study [42]. In the present work, we aimed to determine the possible effect of mutations associated with NS on DNA G4 formation and RNA G4 stability by focusing on the most frequent mutations. Accordingly, the most frequent mutations reported in *NPHS1, NPHS2, CD2AP, ACTN4, WT1, PTPRO, ITGA3* and *ITGB4* genes were selected (Table 2) and induced computationally. Also, the number of predicted PQSs was compared between the wild type and mutated states**.** Interestingly, the number of predicted G4 sequences did not change for all the selected genes except for *NPHS1*. Importantly, the frameshift deletion c. 121_122del in *NPHS1* exon 2 shifted the G4 sequence located 1 bp in proximity leading apparently to a destabilization of the DNA G4 sequence that may potentially affect the transcription of this gene **(S2 Fig.).** Furthermore, we demonstrated that this mutation changed the thermodynamic stability of RNA G4 in this region in by using RNAfold software. Nephrin is one of the major components of the slit membrane that has an important role in maintaining the structure of the podocyte slit membrane, c. 121_122del mutation in *NPHS1* is highly prevalent (>90%) in the Finnish population. The latter mutation causes severe early-onset phenotypes due to total absence of nephrin [[43], [44], [45]]. Our finding proposes a new role for the implication of G-quadruplexes in the resulted phenotype through regulating NPHS1 gene expression. The G4 sequence is located on non-template strand in *NPHS1* gene. And, it is thought that the formation of G4 sequences on the non-template strand increases the final RNA product level due to the formation of co-transcriptional R-loops [46]. We suspect that the upregulation of NPHS1 expression could be facilitated by a G4 ligand, given that the G4 sequence is formed in the non-template strand of the NPHS1 gene [46].Similarly, most of the other mutations in the selected genes affected the RNA G4 stability in their context suggesting that G4s influence the translation or splicing efficiency of glomerular genes in NS patients.

G-rich RNA sequences are commonly believed to adopt G4 structures more easily and have better stability than their DNA G4s. Although RNA G4s are more prevalent in the regulatory regions such as 5′UTR and 3′UTR, many studies demonstrated their presence in ORFs suggesting potential roles in pre-mRNA splicing and translation [47]. Normally, the stabilization of RNA G4s represents a knot slowing-down the splicing or the translation. However, sometimes it could act in an opposite way [48]. For example, G4-forming sequences within 5′- UTRs of FGF2 (fibroblast growth factor 2) and VEGF (vascular endothelial growth factor) mRNAs were demonstrated to stimulate translation. By using on site-directed mutagenesis, the authors suggested that these G4s were part of IRESes (Internal Ribosome Entry Site) that initiate protein synthesis by a non-canonical 5′-cap-independent manner. Although few studies reported the ability of specific RNA G4s to stimulate protein synthesis, exploring the mechanism is still not well understood. In our study, we found that each mutation has its unique case. For example, the severe phenotype observed in patients with NS type 1 harboring the frameshift deletion c. 121_122del in NPHS1, may be explained by the fact that the increase in the thermodynamic stability of RNA G4 decreases the translation efficiency of NPHS1 leading to the absence of expression of this gene. Contrary to this case, although patients with Finnish congenital nephrotic syndrome (FCNS) carrying the mutation c.3325C > T, p.R1109X in NPHS1, present the same severe phenotype, we speculate that the decrease in the thermodynamic stability of the RNA G4 may reduce the affinity of some RNA binding proteins (RBPs) that act to unwind these structures, leading to a decrease in the pre-mRNA splicing efficiency. RNA-binding proteins can have dual roles, acting as either stabilizers for G-quadruplexes or as unwinders, depending on the specific context and cellular conditions [49]. In conclusion, the function of RNA-binding proteins binding G-quadruplexes represents another important factor that needs to be taken in consideration.

Although the effects of G4 motifs during the different processes of gene regulation are always complex and depend on different factors (the location of the G4 sequence and the mutation, the interaction with RNA binding proteins, the difference in motion of ribosome), we suggest that the effect of the mutations on RNA G4 stabilization might contribute to the resulting phenotypes.

### 3.1 Limitations of the study

The use of bioinformatics tools has proven effective for recognizing and describing the potential DNA structures and their involvement in regulating the transcription of target genes. However, mechanistic analysis to fully uncover the role of such sequences in the transcription of the above mentioned genes along with their role in the development of kidney disease still to be done in future studies. Furthermore, we limited our study to mutations known to cause major renal phenotypes and studied their effect on the structure of G4 sequences located within or in the vicinity of these mutations. But we cannot exclude the role of other mutations that might influence the structures of G4 sequences even if present at farther distances. The full description of all mutations and their effects on the entire profile of all G4 sequences in a particular podocyte marker gene requires laborious work and major fund to achieve.

## 4 Conclusion

In conclusion, this study document for the first time the presence of G-quadruplexes in glomerular genes and their potential contribution in the etiology of nephrotic syndrome. The present data provides compelling evidence using bioinformatics analysis demonstrating the presence of stable G-quadruplexes in podocyte-marker genes, and that the most frequent reported genetic mutations in nephrotic patients in the selected genes can alter DNA G4 formation and/or the thermodynamic stability of RNA G4. Further *in-vitro* and *in-vivo* studies are essential to better investigate these findings, which may offer the opportunity to implement novel therapeutic approaches targeting G-quadruplexes in the future.

## 5 Materials and methods

### 5.1 Sequences analyzed and the process of analysis

The FASTA sequences of the complete genes with the regions 1000 base pairs before the TSS were retrieved from UCSC genome browser. These sequences refer to the human RefSeq (assembly GRCh37 (hg19)). G4Hunter web application (http://bioinformatics.cruk.cam.ac.uk/G4Hunter/) was used to identify PQS within promoter regions and complete gene sequences [36]. A threshold of 1.2 and a window size of 25 nucleotides were used as parameters for analysis. The same sequences were re-analyzed using another PQS-predicting tool called QGRS Mapper that searches for PQS according to a pattern-matching sequence [35]. The standard parameters (minimum G-group of 2 and loop size between 0 and 36) were set as default. The highest scoring sequences (indicating those most likely to form G4s) containing 3 ≥ G were documented. The FASTA sequences of the complete genes of *NPHS1* and *PODXL* with the regions 1200 and 1300 base pairs respectively before the TSS were retrieved from UCSC genome browser and re-analyzed by G4Hunter and QGRS Mapper for the prediction of WT1 binding sites.

For the mutations part, the most frequent mutations in NS patients associated with the selected genes were obtained from the literature and OMIM databases. These mutations were created in the genomic sequences by using ChromasPro. Possible overlaps between the predicted PQS and the mutations and their localization were determined by using custom codes in python SNP-locator and SNP-overlap [39]. Then, the overlaps were confirmed by using UCSC genome browser. The wild type genomic sequences and the mutated genomic sequences were re-analyzed by using G4Hunter for their predicted PQS and then compared.

The effect of a mutation on the thermodynamic stability of an RNA G4 was evaluated using RNAfold in the context of the exon or intron containing mutation and their flanking regions (exon and intron). This algorithm allows the determination of the minimum free energy ΔG° (Kcal/mol) by considering the most important thermodynamic parameters which contribute to RNA G4 stability [50].

## Funding

N/A.

## Ethical statement

N/A.

## Author contribution statement

Mona Saad; Cybel Mehawej; Wissam Faour: Conceived and designed the experiments; Performed the experiments; Analyzed and interpreted the data; Contributed reagents, materials, analysis tools or data; Wrote the paper.

## Data availability statement

Data will be made available on request.

## Declaration of competing interest

The authors declare that they have no known competing financial interests or personal relationships that could have appeared to influence the work reported in this paper.
