# Supplementary material for: Analysis of G-quadruplex forming sequences in podocytes-marker genes and their potential roles in inherited glomerular diseases
Source: Heliyon. 2023 Sep 15;9(9):e20233. doi: 10.1016/j.heliyon.2023.e20233 (PMC10559976; doi:10.1016/j.heliyon.2023.e20233)
Supplement: Multimedia component 1 [file mmc1.docx]

**Bioinformatics Analysis of Putative G-Quadruplex Forming Sequences in Podocytes-Marker Genes and Their Potential Roles in Inherited Glomerular Diseases**

**Heliyon Journal**

Mona Saad ^a^, [Cybel Mehawej](https://pubmed.ncbi.nlm.nih.gov/?term=Mehawej+C&cauthor_id=32628964) ^b^, Wissam H. Faour ^a^*

^a^ Gilbert and Rose-Marie Chagoury School of Medicine, Lebanese American University, Byblos, Lebanon.

^b^ Department of Human Genetics, [Gilbert and Rose-Marie Chagoury School of Medicine](http://medicine.lau.edu.lb/?utm_source=lau_email&utm_medium=email&utm_campaign=EmailSig2018), Lebanese American University, Byblos, Lebanon.

* Corresponding author: Wissam H. Faour, PhD; Associate professor of pharmacology; Gilbert & Rose-Marie Chagoury School of Medicine; Lebanese American University; Byblos, Lebanon. P.O. Box 36; wissam.faour@lau.edu.lb

| Gene name | Official symbol | Gene ID | Function |
| --- | --- | --- | --- |
| NPHS1 adhesion molecule, nephrin | *NPHS1* | 4868 | Member of the immunoglobulin family of cell adhesion molecules that functions in the glomerular filtration barrier in the kidney. |
| NPHS2 stomatin family member, podocin | *NPHS2* | 7827 | Regulation of glomerular permeability |
| actinin alpha 4 | *ACTN4* | 81 | Spectrin gene superfamily, actin-binding protein |
| CD2 associated protein | *CD2AP* | 23607 | Scaffolding molecule that regulates the actin cytoskeleton |
| podocalyxin like | *PODXL* | 5420 | Sialomucin protein family, originally identified as an important component of glomerular podocytes. |
| Synaptopodin | *SYNPO* | 11346 | Actin-associated protein that may play a role in actin-based cell shape and motility. |
| WT1 transcription factor | *WT1* | 7490 | Essential role in the normal development of the urogenital system |
| membrane metalloendopeptidase | *MME* | 4311 | Type II transmembrane glycoprotein and a common acute lymphocytic leukemia antigen that is an important cell surface marker in the diagnosis of human acute lymphocytic leukemia (ALL). |
| protein tyrosine phosphatase receptor type O | *PTPRO* | 5800 | PTPRO was demonstrated to regulate glomerular pressure and permselectivity [51][52] |
| vascular endothelial growth factor A | *VEGFA* | 7422 | It encodes a heparin-binding protein. This growth factor induces proliferation and migration of vascular endothelial cells, and is essential for both physiological and pathological angiogenesis. |
| integrin subunit beta 1 | *ITGB1* | 3688 | Membrane receptors involved in cell adhesion and recognition in a variety of processes including embryogenesis, hemostasis, tissue repair, immune response and metastatic diffusion of tumor cells. |
| integrin subunit alpha 3 | *ITGA3* | 3675 | The encoded preproprotein is proteolytically processed to generate light and heavy chains that comprise the alpha 3 subunit. This subunit joins with a beta 1 subunit to form an integrin that interacts with members of the laminin family. The laminin-binding integrin α3β1 is expressed at high levels in lung epithelium and in kidney podocytes. In podocytes, α3β1 associates with the tetraspanin CD151 to maintain a functional filtration barrier. |
| integrin subunit beta 3 | *ITGB3* | 3690 | Integrin beta 3 is found along with the alpha IIb chain in platelets. Integrins are known to participate in cell adhesion as well as cell-surface mediated signaling. |
| integrin subunit alpha V | *ITGAV* | 3685 | This subunit associates with beta 1, beta 3, beta 5, beta 6 and beta 8 subunits. This integrin may regulate angiogenesis and cancer progression. |
| integrin subunit alpha 6 | *ITGA6* | 3655 | This subunit may associate with a beta 1 or beta 4 subunit to form an integrin that interacts with members of the laminin family. The alpha 6 beta 4 integrin may promote tumorigenesis. |
| integrin subunit beta 4 | *ITGB4* | 3691 | Receptor for the laminins, tends to associate with alpha 6 subunit and is likely to play a pivotal role in the biology of invasive carcinoma. It represents a receptor for CD151. |
| CD151 molecule (Raph blood group) | *CD151* | 977 | It is involved in cellular processes including cell adhesion and may regulate integrin trafficking and/or function. |
| KRAS proto-oncogene, GTPase | *KRAS* | 3845 | Small GTPase which is implicated in various malignancies, including lung adenocarcinoma, mucinous adenoma, ductal carcinoma of the pancreas and colorectal carcinoma. |
| HRas proto-oncogene, GTPase | *HRAS* | 3265 | Defects in this gene are implicated in a variety of cancers, including bladder cancer, follicular thyroid cancer, and oral squamous cell carcinoma. |
| MYC proto-oncogene, bHLH transcription factor | *MYC* | 4609 | Role in cell cycle progression, apoptosis and cellular transformation. |
| Wnt family member 1 | *WNT1* | 7471 | Implicated in oncogenesis and in several developmental processes, including regulation of cell fate and patterning during embryogenesis. |
| Wnt family member 2 | *WNT2* | 7472 |  |

**S1 Table** The selected podocytes-marker genes with their official symbols, Gene ID and protein function

| Gene | Highest scoring sequences detected by QGRS Mapper | G-score | Total nb of G4 | Ratio (highly stable G4s/weak G4s) |
| --- | --- | --- | --- | --- |
| *NPHS1* | **GGGCGGGGCGGGAGGG** | **41** | **218** | **0.01** |
|  | **GGGAAGGGAATGGGCTAGGG** | **41** |  |  |
|  | **GGGGAGGGGTCCCGGGGATGGG** | **41** |  |  |
| *NPHS2* | **GGGGCCTGTCGGGGGGTGGGGAGCTGGGG** | **60** | **83** | **0.01** |
| *ACTN4* | **GGGGCTGGGGCAGGACGGCGGGGCTGGGGG** | **56** | **605** | **0.002** |
| *CD2AP* | **GGGGCGGGGTGGGGCGGGG** | **63** | **335** | **0.003** |
| *PODXL* | **GGGGGAGGGGCGGGGGCGGGGGCGGGGG** | **79** | **373** | **0.003** |
| *SYNPO* | **GGGCTGGGCTGGGCTGGG** | **42** | **145** | **0.007** |
| *WT1* | **GGGGCTGGAAGTTGGGGCGGGGGCCGGGG** | **56** | **256** | **0.004** |
| *MME* | **GGGGTAGGGGGTGGGGGGGG** | **61** | **244** | **0.004** |
| *PTPRO* | **GGGGCCTGTCATGGGGTGGGGGGAGGGGGG** | **58** | **616** | **0.002** |
| *VEGFA* | **GGGGGGTGGGGGTTGGGAGCTGGG** | **42** | **179** | **0.01** |
|  | **GGGAAGTGGGGCAAGGGGGGGATAGGG** | **42** |  |  |
| *ITGB1* | **GGGGGCGGGGGCGGGGGCGGGGG** | **84** | **161** | **0.006** |
| *ITGA3* | **GGGGAAGGGGCTGGGGAGGGG** | **62** | **300** | **0.003** |
| *ITGB3* | **GGGAATTGGGACAGGGGAATGGGG** | **42** | **336** | **0.009** |
|  | **GGGCGGGTGGGGGGG** | **42** |  |  |
|  | **GGGAGGGTGGGTGGG** | **42** |  |  |
| *ITGAV* | **GGGCCGCGGGCACTGGGCGCCTCGCTGGG** | **37** | **159** | **0.006** |
| *ITGA6* | **GGGGCGAGAGGGTGGGGAGGGGCGGGG** | **55** | **327** | **0.003** |
| *ITGB4* | **GGGCTGGGCTGGGCAGGG** | **42** | **360** | **0.003** |
| *CD151* | **GGGGCGGGGCGGGGCGGGG** | **63** | **92** | **0.01** |
| *KRAS* | **GGGCGGGCTGGGTGAGAGGGGTCTGCAGGG** | **42** | **118** | **0.009** |
| *HRAS* | **GGGTGGGTGGGGCCGGGCGGGGCCCGCGGG** | **42** | **46** | **0.07** |
|  | **GGGCCTGGGGCTGGGCCTGGG** | **42** |  |  |
|  | **GGGACGGCAGGGCAGTGAGGGAGGCGAGGG** | **42** |  |  |
| *MYC* | **GGGGCGGTACTGGGGGTGGGGACGGGGG** | **58** | **42** | **0.02** |
| *WNT1* | **GGGCCACCGGGCAGGGGGCGGGG** | **40** | **41** | **0.025** |

**S2 Table** The highly stable G4 sequences (containing a minimum of 3 G-tetrads) detected by QGRS Mapper in the genomic sequences are documented here with their G-score

***NPHS1*, NG_051206.1, Chr 19**

Wild type

Intron 1-exon 2-intron 2

gtgag tgggatccca gccttgtacc

cagcgccaag tggcccccat ttcccactta ccttccctag acaaaccctc

tgccctttcc ttgcaccgcg ctgtgtcctc agGCCTGGCG CAGTTGGCGA

TTCCTGCCTC CGTTCCCCGG GGCTTCTGGG CCCTGCCTGA AAACCTGACG

GTGGTGGAGG GGGCCTCAGT GGAGCTGCGT TGTGGGGTCA GCACCCCTGG

CAGTGCGGTG CAATGGGCCA AAGATGGGCT GCTCCTGGGC CCCGACCCCA

GGATCCCAGG CTTCCCGAGG TACCGCCTGG AAGGGGACCC TGCTAGAGgt

aagggatcag agcctgagac cctcagccac cagcggaagc tgcggccctg

acttccggct ccccctgcag

mutation 1: c. 121_122del

gtgag tgggatccca gccttgtacc

cagcgccaag tggcccccat ttcccactta ccttccctag acaaaccctc

tgccctttcc ttgcaccgcg ctgtgtcctc agGCCTGGCG CAGTTGGCGA

TTCCTGCCTC CGTTCCCCGG GGCTTCTGGG CCCTGCCTGA AAACGACG

GTGGTGGAGG GGGCCTCAGT GGAGCTGCGT TGTGGGGTCA GCACCCCTGG

CAGTGCGGTG CAATGGGCCA AAGATGGGCT GCTCCTGGGC CCCGACCCCA

GGATCCCAGG CTTCCCGAGG TACCGCCTGG AAGGGGACCC TGCTAGAGgt

aagggatcag agcctgagac cctcagccac cagcggaagc tgcggccctg

acttccggct ccccctgcag

G4 sequence (wild type): GACGGTGGTGGAGGGGGCCTCAGTGGAG

G4 sequence (mutated): GAAAACGACGGUGGUGGAGGGGGCCUCAGUGGAG

CT: c. 121_122del

**S1 Fig.** Representation of the genomic region (Intron 1-exon 2-intron 2) of NPHS1 gene, the mutation c. 121_122del, with the predicted G4 sequence detected by G4Hunter in wild type and mutated context

|  |  |  | ΔG (kcal/mol) | |  |  |  |  |
| --- | --- | --- | --- | --- | --- | --- | --- | --- |
| Gene | **Mutation** | **RNA G4 Sequence (5'-3')** | **Wild type** | **Mutant** | **Stabilizing effect** | **Phenotype** | **Protein expression** | **Reference** |
| *NPHS1* | c. 121_122del, p.Leu41fs **(a)** | GACGGUGGUGGAGGGGGCCUCAGUGGAG /GAAAACGACGGUGGUGGAGGGGGCCUCAGUGGAG | -163.9 | -164.3 | Stabilizing | Large placenta and severe proteinuria at birth, with nephrotic syndrome appearing during the first weeks of life, generally cause severe early-onset phenotypes | Truncated protein, a total absence of nephrin molecules in the podocyte slit diaphragm | [1, 2] |
|  | c.3325C>T, p.R1109X | GGAUGAAGGUGUGGGGGGAAGUUGAGUGCUG | -120.7 | -119.2 | Destabilizing | Generally cause severe early-onset phenotypes | Truncated protein, absence of nephrin molecules in the podocyte slit diaphragm | [1] |
| *NPHS2* | c.413G>A; p.R138Q | GAAAGGUUUAGUAGUGGGGGUUUGGAG | -1356.14 | -1355.64 | Destabilizing | Renal failure necessitating transplantation | ND, altered function | [3] |
|  | c.686G>A, p.R229Q **(b)** | GCUGGCAGGAACGGUGGGGUUGGUGGGGAUGGACAGGAGGGGUUGG | -1279.9 | -1280.2 | Stabilizing | R229Q homozygotes have a mild phenotype.  The classical phenotype is an early-onset “multidrug-resistant” NS with evolution to end-stage kidney disease and a very low recurrence rate after  renal transplantation | Decreased expression | [4, 5] |
| *CD2AP* | g.47544259_47544260delinsCT; p. P243S **(c)** | GGUUGGGGGUGGGGGGCAG | -846.70 | -846.70 | Inconsequen- tial | - | Decreased expression | [6] |
|  | c.1834C>T, p.R612X **(d)** | GGAAGAGGGGGGGAAUAGGAUUAAGGAAAGAGGG | -729.8 | -729.6 | Destabilizing | Severe elevated proteinuria, progressive deterioration of the glomerular  filtration rate after immunosuppressive therapy. After  renal transplantation, patient growth is still insufficient despite  normal renal function and low steroid dose. | Truncated protein; no CD2AP expression | [7] |
| *ACTN4* | c.763A>G; pK255E **(e)** | GCCCGAGUAGGGCGGGGGGUGGAGGCGUCACUCACUGGG | -2143.4 | -2145.9 | Stabilizing | Mild increase in urine protein excretion, slowly progressive renal dysfunction, and the development of end-stage renal failure in some affected individuals. | Increased expression | [8] |
|  | c.776C>T; p.T259I **(e)** | GCCCGAGUAGGGCGGGGGGUGGAGGCGUCACUCACUGGG | -2143.4 | -2142.4 | Destabilizing | No proteinuria | - | [8] |
|  | c.784T>C; p.S262P **(e)** | GCCCGAGUAGGGCGGGGGGUGGAGGCGUCACUCACUGGG | -2143.4 | -2144.6 | Stabilizing | - | - | [8] |
| *WT1* | c. 1180 C>T; p.R394W **(f)** | GGGGAUCUGGAGUGUGAAUGGGAGUGG | -1281.84 | -1281.14 | Destabilizing | Severe urogenital aberrations, renal failure, pseudohermaphroditism, and Wilms' tumor | dominant-negative or gain-of-function fashion, loss of DNA binding | [9] |
| *PTPRO* | c.2627+1G>T; p.E854_W876del **(g)** | GGAGAUGCUGGGUUGGCAUGGGGGCUCAGGCCUG | -2606.9 | -2606.2 | Destabilizing | Focal segmental glomerulosclerosis and mild focal tubulointerstitial fibrosis and atrophy, diffuse foot process effacement and widespread attenuation of the glomerular basement membranes without thickening, podocytes swollen and vacuolated | Shortened protein, non altered expression | [10] |
|  | c.2745+1G>A **(h)** | GGAAAGGGCCUGGGAAUUGGGGGGUUUGUGUUGCGGUCAUG | -2659.4 | -2655.3 | Destabilizing | Global sclerosis of 3 of 38 glomeruli with narrow zones of interstitial fibrosis and tubular atrophy around the globally sclerosed glomeruli, diffuse foot process fusion and extensive microvillus transformation of the podocytes. Another patient presented a more severe phenotype. | Degradation of mRNA, complete absence of PTPRO | [10] |
| *ITGA3* | c.1883G>C; p.R628P | GGAACUGGACCUGGGGGGUGGCCGGAGGUGUGAAGG  and GGCCAAGGGUGGGACGGGGCCUCAUUAACUGGCAGGGUGGGGGCGGGGCCUCAUGGCAAGGCGAG | -236.8 | -238.30 | Stabilizing | Demise: 19 months of multiorgan failure related to infection | Loss of integrin | [11] |
|  | c.1387C>T; p.R463W **(i)** | GGGAGGCUAGGAGGGGCUGCAG | -437.9 | -437.9 | Inconsequential | Demise: 6.5 months of respiratory infection | Increased expression | [11] |
| *ITGB4* | c.3841C>A; p.R1281W **(j)** | GGCGCGCAACGGGGCCGGCUGGGGGCCUGAGCGGGAGG | -532.2 | -531.1 | Destabilizing | Non-lethal phenotype, at age  3 months: blistering of the skin, as well as  severe nephrotic syndrome | Decreased expression | [12, 13] |

**(a)** The protein resulting from the substitution of glutamine for arginine is retained in the endoplasmic reticulum (ER) and loses its ability to recruit nephrin in lipid rafts [14].

**(b)** The resulting podocin has a significant decreased binding to nephrin *in vitro* [5].

**(c)** The length of the genomic sequence uploaded is larger than 10 000 bp, so the region exon 7-intron 7-exon8-intron 8 was used as input.

**(d)** The length of the genomic sequence uploaded is larger than 10 000 bp, so the region intron 16-exon 17 was used as input. The mutation resulted in a disturbed interaction with F-actin.

**(e)** *In vitro*, mutant α-actinin-4 binds filamentous actin (F-actin) more strongly than does wild-type α-actinin-4 [8].

**(f)** The length of the genomic sequence uploaded is larger than 10 000 bp, so the region intron 6-exon 7 was used as input.
This mutation converts 394Arg to Trp within the conserved third zinc finger domain of WT1. It alters the DNA sequence recognition [9].
Their results show that the zinc fingers bind in the major groove of B-DNA and that most of the contacts are made with the guanine-rich strand.

**(g)** This mutation causes skipping of the evolutionarily conserved exon 16 (p.Glu854_Trp876del) at the RNA level.

**(h)** The length of the genomic sequence uploaded is larger than 10 000 bp, so the region exon 18-intron 18 was used as input.
This mutation causes skipping of exon 19, and this introduces a premature stop codon at the very beginning of exon 20 (p.Asn888Lysfs*3) and causes degradation of mRNA via nonsense-mediated decay.

**(i)** The mutation altered the conformation of the extracellular β-propeller domain of the integrin α3 subunit preventing correct processing of N-linked oligosaccharides, heterodimerization with β1 integrin and maturation through cleavage into heavy and light chains in the Golgi.

**(j)** This mutation renders β4 unable to interact with plectin and prevents the localization of plectin in hemidesmosomes.

**S3 Table** Table illustrating the mutations selected, the RNA G4 sequence implicated, the strand, the minimum free energy ΔG° (Kcal/mol) in wild type and mutant context, the effect on the RNA G4 stability, the phenotype, protein expression, the distance between RNA G4 sequence-mutation and the reference
